# Reviewed article: Cordeiro DC, Aguiar EB, Luiz RR, Sacramento DS, Paolino PLW, Gonçalves MJF, Trajman A. Effect of successive tuberculin skin test readings on healthcare professionals’ proficiency: an operational observational study in five high-tuberculosis-burden Brazilian municipalities, 2023-2024. Epidemiol Serv Saude. 2025:34;e20240795

**DOI:** 10.1590/S2237-96222025v34e20240795.b

**Published:** 2025-10-31

**Authors:** Iukary Oliveira Takenami

**Affiliations:** 1 Universidade Federal do Vale do São Francisco, Petrolina, PE, Brazil Universidade Federal do Vale do São Francisco Petrolina PE Brazil

## Peer review B

## Questionnaire

Does the manuscript contain new and significant information to justify publication? Yes

Does the Abstract (Summary) clearly and accurately describe the content of the article? No

Is the problem significant and concisely stated? No

Are the methods described comprehensively? No

Are the interpretations and conclusions justified by the results? No

Is adequate reference made to other work in the field? No

## Manuscript Structure

Length of article is: Too short

Number of tables is: Adequate

Number of figures is: Adequate

Please state any conflict(s) of interest that you have in relation to the review of this paper. None

Interest: Good

Quality: Below Average

Originality: Good

Overall: Below Average

Recommendation: Reject

## Comments

O resumo segue as seções estruturais adequadas, mas, na conclusão, seria mais apropriado incluir uma ressalva, como: “Esses achados sugerem a possibilidade de revisar as recomendações, mas estudos adicionais são necessários para validar essa proposta.” Além disso, o título poderia ser mais preciso. Embora o resumo mencione o impacto do número de leituras, os resultados indicam que esse fator não teve influência. Ajustar o título para refletir melhor essa conclusão evitaria um viés na interpretação inicial do leitor.

Introdução: a introdução apresenta um tema relevante, mas precisa de ajustes conceituais e estruturais para fortalecer a argumentação. Alguns conceitos que eu sugiro rever, pois são equivocados ou representam imprecisões científicas:

- afirmação de que “23% estão infectados pela tuberculose” deve ser reformulada para “23% estão infectados pelo Mycobacterium tuberculosis”;

- sugiro esclarecer melhor a diferença entre infecção latente e doença ativa e adotar uma terminologia mais precisa;

- termo “tratamento da tuberculose latente” é preferível a “tratamento preventivo da tuberculose”.

- a introdução faz pelo menos duas menções a “dados não publicados”, o que pode comprometer a força do argumento. Caso esses dados sejam essenciais, é necessário fornecer mais informações sobre sua origem ou justificar sua inclusão.

- algumas frases são longas e complexas, dificultando a leitura. Recomenda-se reestruturar a introdução para melhorar a fluidez e a clareza da argumentação;

- a transição para a justificativa do estudo poderia ser mais explícita. Seria útil conectar de forma mais direta a baixa oferta do exame, a necessidade de capacitação e o impacto esperado na ampliação do tratamento da tuberculose latente;

- embora a introdução cite várias referências, algumas afirmações poderiam ser melhor embasadas. Em particular, a alegação sobre a inviabilidade da implantação do IGRA no SUS requer maior fundamentação, pois se trata de um ponto crítico na discussão sobre diagnóstico da tuberculose latente;

- objetivo poderia ser formulado de maneira mais enfática e clara, destacando explicitamente que o estudo avalia o impacto do número de leituras na acurácia dos profissionais capacitados.

Material e métodos:

- o critério de seleção dos municípios não foi claramente explicado. Foram escolhidos apenas por serem de alta incidência ou outros fatores, como a disponibilidade de profissionais, também influenciaram a seleção?

- a frase “A população do estudo consistiu em profissionais de saúde capacitados com o protocolo oficial com algumas simplificações...” deveria especificar quais foram essas simplificações e justificar sua necessidade.

- a metodologia menciona que as capacitações foram realizadas por enfermeiras experientes, mas não detalha o critério de experiência utilizado (tempo de prática? treinamentos prévios?

- o uso de salsichas para a aplicação da tuberculina e modelos de silicone para a leitura do endurado é uma estratégia inovadora, mas carece de embasamento mais sólido. Embora os autores citem alguns artigos, é essencial esclarecer se essas técnicas foram previamente validadas. Existem estudos que comprovam a equivalência desses métodos em relação à prática em pacientes reais? Caso contrário, a confiabilidade desses modelos como ferramenta de capacitação permanece incerta.

- O número de leituras realizadas por profissional não foi padronizado, o que pode gerar viés. Se um profissional realizou muitas leituras e outro poucas, como isso foi controlado na análise?

- O estudo menciona que um esforço foi feito para alcançar 100 leituras por município, mas nem sempre foi possível. Essa variação pode impactar a análise e deveria ser discutida como uma limitação.

- Não há uma descrição detalhada de como possíveis viéses foram controlados. Alguns pontos a serem considerados: i) o efeito da experiência prévia dos profissionais nas leituras foi analisado como um fator de confusão? ii) estudo considera alguma possível tendência dos profissionais a arredondar medidas para determinados valores?

- Os autores mencionam que dividiram os participantes em com e sem experiência prévia. Quais critérios foram considerados como experiência previa?

- Em relação ao CEP, citar o CAAE.

Resultados:

- há um grande desequilíbrio na distribuição dos profissionais capacitados entre os municípios (Manaus teve 108 capacitados, enquanto Porto Alegre teve apenas 12). Isso pode ter gerado viés na análise dos resultados e dificultar a generalização dos achados. A justificativa para essa disparidade não é mencionada. Foi por falta de adesão dos municípios ou dificuldades operacionais? Como isso pode ter impactado os resultados?

- o número de leituras realizadas por cada profissional variou amplamente (de 5 a 122). Profissionais que fizeram poucas leituras podem não ter tido tempo suficiente para desenvolver habilidades, enquanto aqueles que realizaram muitas podem ter apresentado melhora ao longo do tempo. A média de 35,3 leituras por profissional pode mascarar essa grande variação. Uma análise estratificada (por faixas de leituras realizadas) poderia ajudar a entender melhor a curva de aprendizado.

- a média das diferenças entre as leituras dos profissionais capacitados e os multiplicadores foi próxima de zero (0,01 mm ± 0,70 mm), o que sugere um bom desempenho geral. No entanto, a variabilidade (± 0,70 mm) pode ser relevante para leituras próximas ao ponto de corte, especialmente em casos marginais.

- não houve tendência de redução das diferenças ao longo do tempo. Isso pode indicar que a capacitação inicial já foi suficiente, mas também pode sugerir que não houve aprendizado progressivo. Uma análise mais detalhada sobre a evolução das leituras individuais poderia esclarecer isso.

- a concordância geral foi alta (Kappa ponderado 0,864). NO entanto, a menor concordância na faixa de 5-9 mm é preocupante, pois é justamente nessa faixa que a definição de positividade pode ser mais incerta. Esse resultado sugere que profissionais podem ter dificuldades na diferenciação entre leituras limítrofes.

A discussão traz pontos interessantes sobre a eficácia da capacitação com um número reduzido de leituras, mas poderia ser mais crítica ao considerar: i) a possibilidade de melhora com mais leituras, algo que não foi completamente descartado; ii)a relevância da concordância, especialmente em leituras limítrofes; iii) a necessidade de mais evidências antes de recomendar mudanças na diretriz nacional; iv) desafios práticos de implementação de um protocolo reduzido sem comprometer a qualidade das leituras. Se a intenção é sustentar uma mudança na recomendação oficial, o estudo precisaria de mais dados, incluindo um acompanhamento a longo prazo e comparações diretas com diferentes esquemas de capacitação.

